# APRIL-driven BCMA/TACI dual-targeted ligand–drug conjugates for selective and potent therapy of multiple myeloma

**DOI:** 10.1016/j.apsb.2026.02.001

**Published:** 2026-02-06

**Authors:** Linling Zhou, Yanping Sun, Jie Bi, Jiaqi Zhao, Yinli Xia, Jun He, Jisheng Wang, Liqiang Pan

**Affiliations:** a School of Pharmacy and Department of Pharmacy, The Second Affiliated Hospital, Zhejiang University, Hangzhou 310058, China; b School of pharmacy, Hangzhou Medical College, Hangzhou 311399, China; c The Third Hospital of Mianyang (Sichuan Mental Health center), Mianyang 612000, China

**Keywords:** Multiple myeloma, Ligand‒drug conjugate, Antibody–drug conjugate, Immunotherapy, B-Cell maturation antigen (BCMA), A proliferation-inducing ligand (APRIL), Cancer therapy, Dual targeting therapy

## Abstract

B-cell maturation antigen (BCMA) is highly expressed on malignant plasma cells and has emerged as an ideal therapeutic target in multiple myeloma (MM). However, the efficacy of BCMA-targeted therapies is often limited by antigen downregulation and tumor escape. Here, we report the design of a dual-targeted ligand–drug conjugate (LDC) leveraging a proliferation-inducing ligand (APRIL), the natural ligand for both BCMA and transmembrane activator and calcium-modulator and cyclophilin ligand interactor (TACI). This dual-targeting strategy enables enhanced binding breadth and mitigates antigen-loss-mediated resistance. Compared to conventional antibodies, APRIL-based fusion proteins (APRILFc) exhibited markedly faster internalization in MM cells (∼25% within 30 min), facilitating efficient intracellular drug delivery. LDCs conjugated with monomethyl auristatin E (MMAE) or SN-38 demonstrated potent cytotoxicity across diverse MM cell lines, outperforming corresponding antibody–drug conjugates (ADCs). Notably, LDCs maintained strong antitumor activity in BCMA-knockout models, highlighting their capacity to overcome BCMA escape. In both subcutaneous and intratibial MM mouse models, LDC-MMAE significantly suppressed tumor growth, including in the context of BCMA shedding. These findings establish APRIL-based LDCs as a promising, mechanistically distinct modality for MM therapy, capable of addressing key limitations of current BCMA-targeted approaches.

## Graphical abstract

The authors engineered a ligand‒drug conjugate (LDC) leveraging natural APRIL ligand for dual BCMA/TACI targeting exhibit accelerated internalization and superior cytotoxicity over anti-BCMA ADCs. This mechanistically distinct strategy addresses key limitations of current BCMA-targeted approaches.

**Figure:**
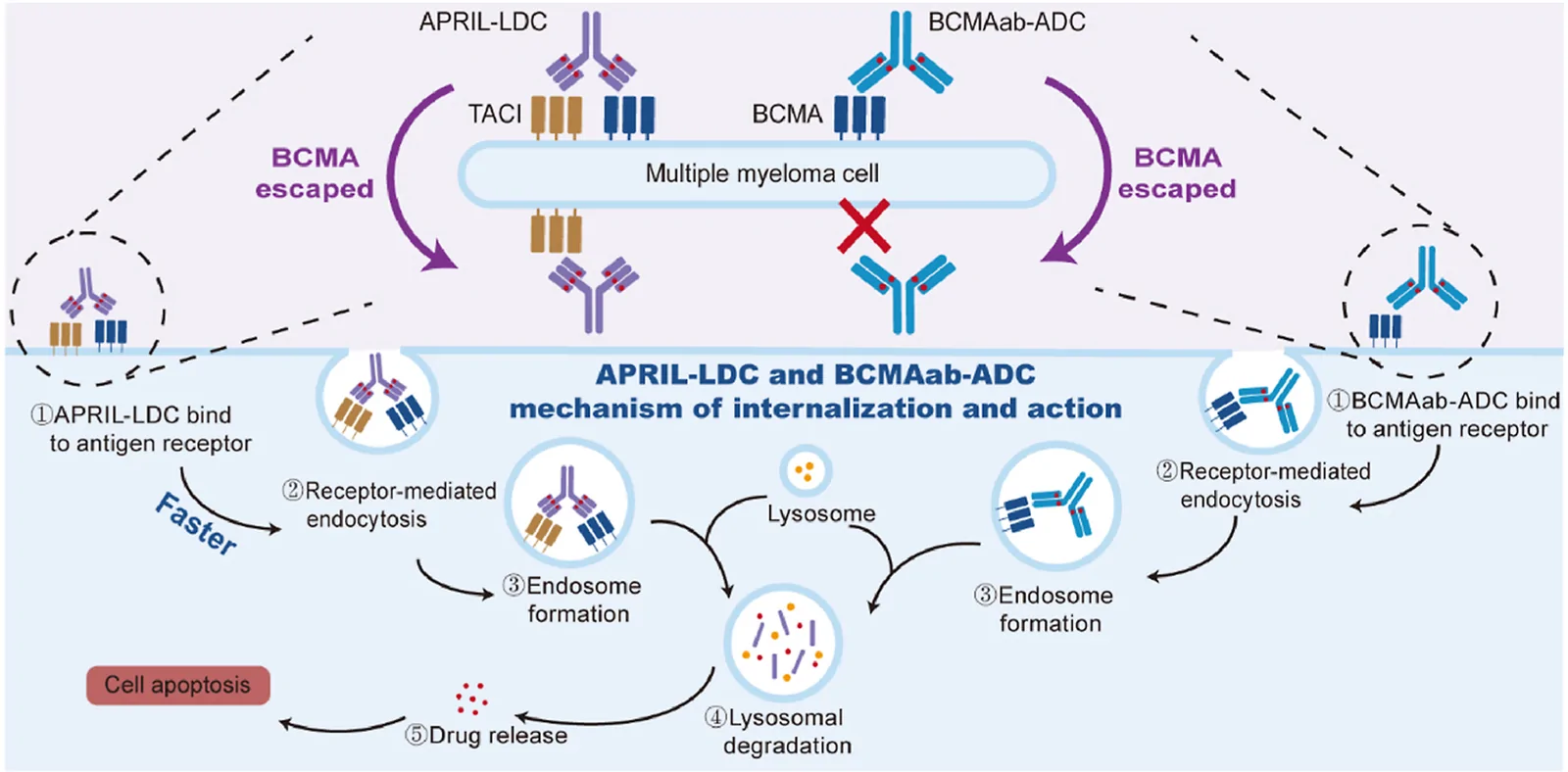

## 1 Introduction

Multiple myeloma (MM) is a hematologic malignancy characterized by the clonal proliferation of neoplastic plasma cells and is the second most common cancer of the blood after lymphoma^1^^,^^2^. It accounts for approximately 1% of all cancers and 2% of global cancer-related mortality^3^^,^^4^. In patients with MM, uncontrolled proliferation of plasma cells in the bone marrow drives excessive production of aberrant immunoglobulins^5^^,^^6^, causing hallmark clinical manifestations including hypercalcemia, renal impairment, anemia, and bone lesions^7^. Recent decades have witnessed the approval of multiple therapeutic regimens such as autologous stem cell transplantation (ASCT)^8^, proteasome inhibitors (PIs)^9^^,^^10^, and immunomodulatory imide drugs (IMiDs)^11^. While most patients attain objective responses, MM remains largely incurable, with the majority eventually developing refractory disease, underscoring the urgent need for novel therapeutic strategies^12^^,^^13^. Currently, several target antigens for MM immunotherapy, such as CD38 and SLAMF7^2^, have been extensively studied. However, their expression in other normal tissues poses the risk of “on-target, off-tumor” toxicity^14^^,^^15^, underscoring the need for more selective therapeutic targets.

BCMA, a type III transmembrane glycoprotein belonging to the tumor necrosis factor receptor superfamily, also known as TNFRSF-17^16^, is predominantly expressed on terminally differentiated B cells and plasma cells, and is expressed on almost all multiple myeloma cells^17^. Notably, elevated BCMA mRNA levels are associated with disease progression, establishing BCMA as both a prognostic biomarker and a compelling therapeutic target for MM^18^, ^19^, ^20^.

Despite the approval of multiple BCMA-targeted immunotherapies, such as the antibody‒drug conjugate (ADC) Belantamab mafodotin^21^^,^^22^, which provides important therapeutic options for patients with multiple myeloma. BCMA antigen downregulation and shedding limit their efficacy, resulting in disease relapse^23^^,^^24^. To address this challenge, dual-antigen targeting strategies have emerged to enhance tumor recognition and minimize antigen escape. TACI, another member of the TNFRSF family, exhibits highly restricted expression predominantly on plasma cells and shows increased expression levels in most myeloma cells^25^^,^^26^. APRIL, primarily secreted by myeloid cells, is a natural high-affinity ligand for both BCMA and TACI^27^, ^28^, ^29^. Despite becoming emerging areas of drug development, bispecific antibodies and dual-targeting ADC platforms, which typically utilize engineered antibodies to engage two tumor antigens simultaneously, carry the potential for heightened off-target toxicity and are inherently complex to manufacture.

In this study, we developed a novel ligand–drug conjugate (LDC) platform that harnesses APRIL's inherent bispecificity to simultaneously target BCMA and TACI, enabling efficient and selective intracellular delivery of cytotoxic agents to MM cells. Engineered APRIL-based ligand fusion proteins exhibited superior binding affinity and receptor clustering capability compared to monoclonal antibodies, significantly enhancing internalization kinetics. In contrast to conventional BCMA-ADCs, the LDC induced rapid apoptosis and potent cytotoxicity across MM cell lines with heterogeneous BCMA/TACI expression. Moreover, the LDCs effectively inhibited tumor progression in multiple *in vivo* models, including disseminated MM, subcutaneous xenografts, and BCMA-knockout escape models, highlighting their therapeutic potential in overcoming antigen loss-mediated resistance.

## 2 Materials and methods

### 2.1 Animals

Healthy specific pathogen-free female mice, aged 5–6 weeks, belonging to the NOD/SCID or NCG strain, were obtained from GemPharmatech Co., Ltd. (Nanjing, China). Animal experiments were approved by the Laboratory Animal Welfare and Ethics Committee of Zhejiang University (reference number 31067) and were performed in accordance with the National Institutes of Health Guide for the Care and Use of Laboratory Animals.

### 2.2 Cell lines and cell culture

The Comprehensive AIDS Research Center (Tsinghua University) generously provided human embryonic kidney 293 F cells (HEK293F), cultured in suspension in SMM 293-TII medium (Sino Biological, cat# M293TII) supplemented with 0.5% fetal bovine serum (FBS) (Gibco, cat# A5669701). Multiple myeloma cell lines MM.1S (RRID: CVCL_8792) and H929 (RRID: CVCL_1600) were kindly gifted by Prof. Zhen Cai from the First Affiliated Hospital of Zhejiang University. RPMI8226 (RRID: CVCL_0014), L363 (RRID: CVCL_1357), and Daudi (RRID: CVCL_0008) cells were kindly provided by AnsonBio (Shanghai) Pharmaceutical Technology Co., Ltd. All myeloma cell lines were maintained in RPMI 1640 medium (Gibco, cat# C1187550BT) containing 10% FBS. The human erythroleukemia cell line K562 was obtained from the American Type Culture Collection (ATCC, cat# CCL-243) and cultured in RPMI 1640 medium (Gibco) with 10% FBS. MM.1S-Luc cells (cat# JY257) were purchased from Jinyuan (Shanghai) Biotechnology Co., Ltd., and maintained in RPMI 1640 (Gibco) supplemented with 10% FBS and 1.5 μg/mL puromycin. All cell lines were contamination free. The cell lines were cultured in a humidified chamber at 37 °C with 5% CO_2_.

### 2.3 Construction and expression of APRILFc fusion proteins

To construct the APRILFc fusion protein, the sequence of mAPRIL (PDB ID: 1XU2) was engineered into a tandem trimeric configuration using a G_4_SG_4_S linker and fused at the C-terminus to the Fc domain of human IgG1. The synthesized gene cassette was cloned into the pcDNA3.1(+) expression vector. To enable site-specific conjugation of vc-SN38 and vc-MMAE, we introduced cysteine mutations at a distal loop region of the APRIL binding domain, strategically selected based on structural analysis to minimize interference with ligand‒receptor interactions. All gene sequences were commercially synthesized by GenScript (Nanjing, China) to ensure codon optimization and sequence fidelity. The fusion protein was produced by transient transfection of HEK293F cells using polyethylenimine (PEI, YEASEN, cat# 40816ES02) as the transfection reagent. Until HEK293F cells reached the appropriate density, plasmid DNA and PEI were sterilized by filtration through a 0.22 μm filter and diluted to 0.05 and 0.1 mg/mL in SMM 293-TII Expression Medium. The diluted DNA and PEI were then mixed at equal volumes and incubated at room temperature for 15 min to allow complex formation. The DNA/PEI mixture was subsequently added to HEK293F cells in the flask, followed by incubation at 37 °C for 3–5 days. After transfection, the culture supernatant was collected and purified using a HiTrap Protein A HP affinity chromatography column on an AKTA pure 25 system. The purity of the eluted protein was assessed by SDS-PAGE, and the purified samples were stored at −80 °C for further use.

### 2.4 Flow-cytometric analysis

The binding of APRILFc fusion protein to native BCMA or TACI antigens on the surface of tumor cells was assessed using flow cytometry. Cells (5 × 10^6^ per tube) were collected and incubated with 300 to 1.2 nmol/L of the test protein (diluted in a 3-fold concentration gradient) for 1 h at 4 °C. After washing three times with PBS, FITC-conjugated secondary antibody (A0556, Beyotime Biotechnology, Shanghai) was added, and the cells were incubated at 4 °C in the dark for an additional hour. Following a final wash, labeled cells were analyzed using the ACEA NovoCyteTM (ACEA Biosciences, Hangzhou, China). The expression levels of BCMA and TACI on different tumor cell lines were quantified using BD Quantibrite™ PE Phycoerythrin Fluorescence Quantitation Kit (BD Biosciences, 340495). Cells were incubated with PE-anti human BCMA antibody (BioLegend, 357504) or PE-anti human TACI antibody (BioLegend, 311906). Fluorescence intensity was measured using an ACEA NovoCyteTM, and the mean fluorescence intensity (MFI) values were converted to antibody-binding capacity (ABC) values per cell.

### 2.5 LDC production

The APRILFc-S107C protein was adjusted to a concentration of 1–2 mg/mL and reduced by 15-fold molar equivalents of Tris(2-carboxyethyl)phosphine hydrochloride (TCEP, cat# C4706, Merck, Germany) at 4 °C with overnight rotation. The reduced APRILFc-S107C was conjugated with 6 molar equivalents of mc-vc-PAB-MMAE (cat# SET0201, Levena Biopharma, Nanjing, China) at 25 °C for 2 h under constant rotation. Following conjugation, the APRILFc-MMAE was buffer exchanged into PBS and unconjugated payloads were removed by centrifugal ultrafiltration (Amicon Ultra-0.5 mL 30 KD, Millipore). The conjugation efficiency of the protein–toxin complex was analyzed by SDS-PAGE, and the final product was aliquoted and stored at −80 °C for subsequent experiments.

### 2.6 CRISPR gene editing of cell lines

The LentiCRISPRv2 system was used to knockout the BCMA of RPMI8226 cells to generate RPMI8226-BCMA-KO cells. The lentiCRISPR v2 was a gift from Feng Zhang (Addgene plasmid # 52961; http://n2t.net/addgene:52961; RRID:Addgene_52961). The BCMA-targeting CRISPR sgRNA plasmid was mixed with lentiviral packaging plasmids pMD2.G and psPAX2 at a molar ratio of 5:2:3 in culture medium, followed by the addition of polyethylenimine (PEI) to form transfection complexes. After 15 min of incubation at room temperature, the plasmid/PEI mixture was co-transfected into HEK293T cells to produce lentiviral particles. Viral supernatant was collected 48 h post-transfection, filtered through a 0.45 μm membrane, and stored at −80 °C for subsequent experiments. For lentiviral transduction, RPMI8226 cells (5 × 10^6^ cells in the logarithmic growth phase) were incubated with viral supernatant supplemented with 7 μg/mL polybrene at 37 °C for 24 h, after which the medium was replaced with fresh culture medium. At 48 h post-transduction, cells were selected with 1.5 μg/mL puromycin for 7 days to enrich CRISPR/Cas9-edited populations. BCMA-negative RPMI8226 cells were subsequently isolated by fluorescence-activated cell sorting (FACS) to establish a clonal BCMA-KO cell line. The following sgRNA sequences were used: BCMA (TNFRSF17): GGUGUGACCAAUUCAGUGAA, UAUUAAGCUCAGUCCCAAAC, UCGUGCUAAUGUUUUUGCTA.

### 2.7 Confocal microscopy

APRILFc was fluorescently labeled with Cy5-SE (TargetMol, Boston, USA). Free CY5 dye was removed by ultrafiltration. RPMI8226 cells (1 × 10^5^ cells/well) were seeded into glass-bottom dishes 24 h prior to treatment and incubated with 10 μg/mL APRILFc-Cy5 at 37 °C for 1, 3, or 12 h. Cells were fixed with 4% paraformaldehyde for 15 min, permeabilized with 0.1% Triton X-100 and 0.2% BSA in PBS for 10 min, and blocked with 2% BSA in PBS for 30 min. For lysosomal localization analysis, cells were incubated with anti-LAMP1 primary antibody (ab278046, abcam, Shanghai) for 30 min, followed by a secondary stain with anti-rabbit IgG (H + L)-Cy3 antibody (Beyotime Biotechnology, Shanghai) or an additional 30 min. Nucleus were counterstained with DAPI (Beyotime Biotechnology, Shanghai) for 10 min. Between each staining step, cells were washed 4‒5 times with PBS. Confocal images were acquired using an OLYMPUS FV3000 high-resolution confocal microscope (Olympus, Tokyo, Japan).

### 2.8 Internalization assay

To quantify internalization of APRILFc and anti-BCMA antibody by flow cytometry, the cells were collected (5 × 10^6^/tube) and incubated with 5 μg/mL APRILFc or anti-BCMA antibody at 4 °C for 30 min. The cells were partitioned into two groups, the non-internalized control group was maintained at 4 °C, while experimental groups were transferred to 37 °C and incubated for varying durations. Cells were then stained with anti-human IgG FITC antibody at 4 °C for 30 min in the dark. After two additional PBS washes, the mean fluorescence intensity (MFI) of stained cells was measured by ACEA NovoCyteTM and analyzed using FlowJo software. The percentage of internalized protein was calculated using the following Eq. (1):

(1) $$\text{Internalization}\;\text{efficiency}\;\left(\%\right)=\left[\left({\text{MFI}}_{4\;{}^{\circ}C}‒{\text{MFI}}_{37\;{}^{\circ}C}\right)\;/\;{\text{MFI}}_{4\;{}^{\circ}C}\right]\times 100$$

To monitor protein internalization in real time, APRILFc and anti-BCMA antibody were labeled with the pH-sensitive fluorescent dye Incucyte® Fabfluor-pH (Sartorius, Germany). Tumor cells (2 × 10^4^ cells/well) were seeded into a 96-well plate and treated with Fabfluor-labeled proteins at final concentrations of 50 or 10 nmol/L. Live-cell imaging was performed using the Incucyte® Live-Cell Analysis System (Sartorius, Germany), with phase-contrast and fluorescence images acquired every hour over a 36-h period. The integrated red fluorescence area per well was quantified automatically by Incucyte® software, and data were normalized to baseline fluorescence for kinetic analysis of internalization dynamics.

### 2.9 *In vitro* cell proliferation assay

To evaluate the cytotoxic effects of LDCs and ADCs, tumor cells (5 × 10^3^ cells/well) were seeded into 96-well plates and treated with increasing concentrations (0, 0.2, 0.4, 0.8, 1.6, 3.1, 6.3, 12.5, 25, 50 nmol/L) of LDCs or ADCs for 72 h. Following treatment, 50 μL of CCK-8 reagent (DOJINDO, Japan) diluted in culture medium was added to each well to achieve a final concentration of 10% (*v*/*v*), and the plate was incubated at 37 °C for 2 h. Absorbance was measured at 450 nm using a Bio-Rad Model 680 microplate reader. Data were analyzed with GraphPad Prism software, and cell viability was expressed as the percentage of growth inhibition relative to untreated controls.

### 2.10 Detection of apoptosis and cell cycle arrest *in vitro*

RPMI8226 cells were seeded at a density of 5 × 10^4^ cells/well into12-well plates and exposed to varying concentrations of LDCs or ADCs for 12 to 72 h, and the untreated cells served as controls. For apoptosis analysis, cells were harvested, washed with PBS, and stained with 5 μL Annexin V-APC and 5 μL propidium iodide (PI) (DOJINDO, Japan) for 15 min at room temperature in the dark. Apoptotic cell populations were quantified by flow cytometry, distinguishing early apoptotic (Annexin V^+^/PI^−^) and late apoptotic (Annexin V^+^/PI^+^) cells. For cell cycle arrest analysis, cells treated for 12 or 24 h were fixed overnight in 70% ethanol at 4 °C. After fixation, cells were washed with PBS and stained with PI solution containing RNase A (Yeasen, Shanghai) for 30 min at room temperature in the dark. Cell cycle distribution (G0/G1, S, and G2/M phases) was analyzed by flow cytometry using ACEA NovoExpress software.

### 2.11 Antitumor efficacy of LDC *in vivo*

For the bioluminescent MM liquid model, female NCG mice aged 5‒6 weeks were challenged with an i.v. injection of 5 × 10^6^ MM.1S-Luc cells. 20 days post-inoculation, mice were injected i.v. With 100 μL PBS, APRILFc or APRILFc-MMAE twice weekly. Bioluminescence imaging was performed every 3‒5 days, and tumor burden was quantified as mean radiant efficiency (photons/s/cm^2^/sr) per mouse. The mice were euthanized *via* CO_2_ asphyxiation at the end of the experiment.

For the subcutaneous model, 5 × 10^6^ RPMI8226 cells were injected subcutaneously into the right flank of each NOD/SCID mouse, 5 × 10^6^ RPMI8226-BCMA-KO cells were injected subcutaneously into the right flank of each NCG mouse. When tumor volumes reached approximately 80 mm^3^, the mice were randomly allocated to three groups (*n* = 5 per group) and injected intravenously with 100 μL PBS, APRILFc or APRILFc-MMAE every four days for a total of four injections. Tumor dimensions were measured every 3 days using calipers, and tumor volume was calculated as Eq. (2):

(2) $$V=0.5\times \text{Length}\times {\text{Width}}^{2}$$

At the end of experiment, the mice were euthanized *via* CO_2_ asphyxiation. Major organs, including the heart, liver, spleen, and lungs were harvested and fixed in 4% paraformaldehyde for hematoxylin‒eosin (H&E) staining.

### 2.12 Statistical analysis

Graph generation and statistical analysis were performed with GraphPad Prism V.9 software. All data are presented as mean ± standard deviation (SD). Statistical differences between mean values were determined using unpaired two-tailed Student's *t*-test or one-way analysis of variance with Dunnett's post-test analysis as indicated in Figure legends, ∗*P* < 0.05, ∗∗*P* < 0.01, ∗∗∗*P* < 0.001. Survival curves were analyzed by log-rank test. In all cases, a significant result was defined as a *P* < 0.05.

## 3 Results and discussion

### 3.1 Generation and characterization of APRIL-LDC

**Figure 1:**
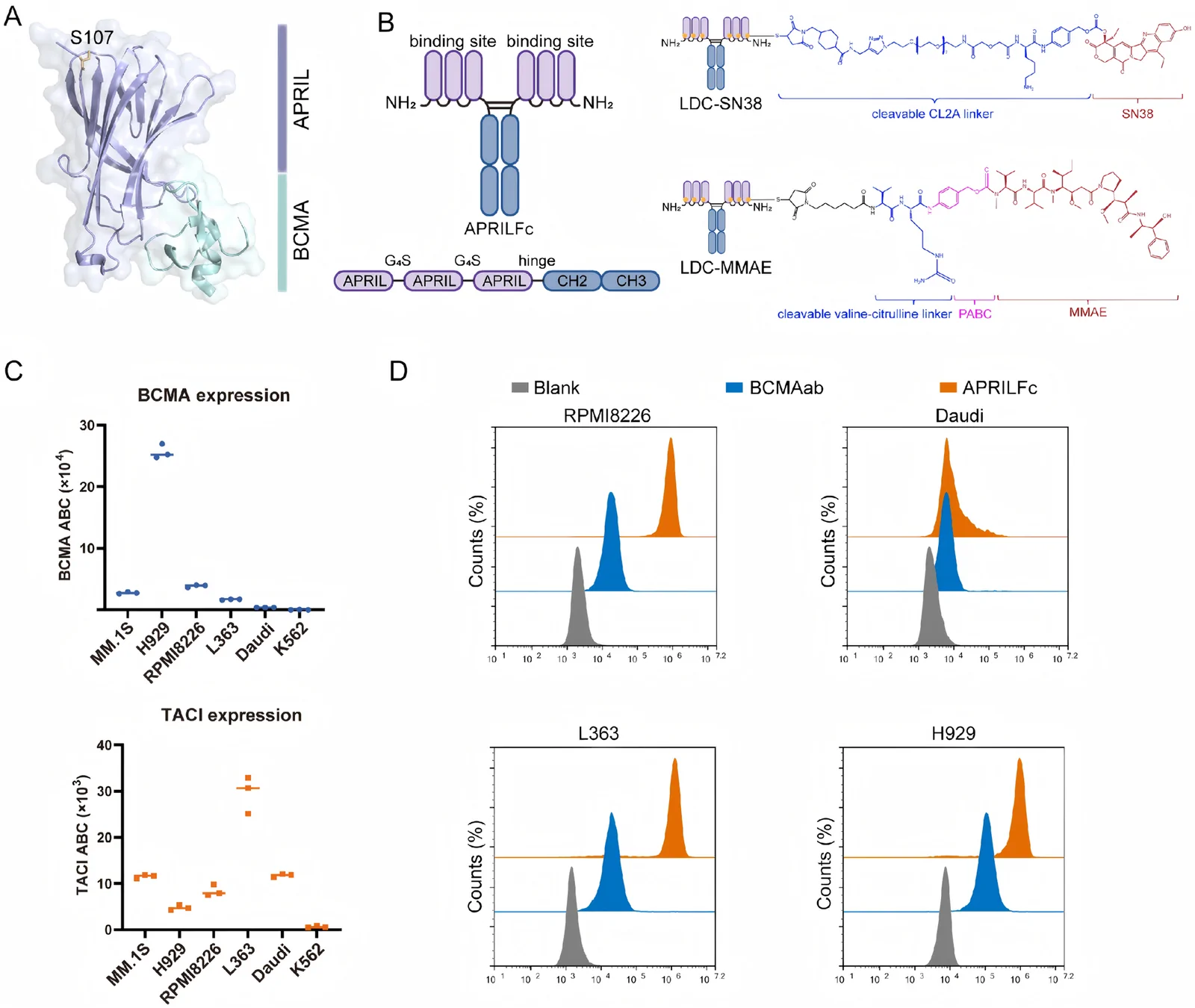
Rational design and expression of APRIL-based ligand‒drug conjugate (LDC). (A) The crystal structure of APRIL bound to BCMA (PDB: 1XU2). (B) Schematic representation of APRIL-LDC design. (C) Quantitative FACS analysis of BCMA and TACI antigen expression in different multiple myeloma cell lines by Quantibrite™ PE Phycoerythrin Fluorescence Quantitation Kit. The ordinate label “ABC” denotes antibody-binding capacity. (D) Comparative binding affinity of APRILFc and BCMAab for different cell lines (RPMI8226, Daudi, L363, H929).

To address BCMA antigen escape, we developed a ligand‒drug conjugate (LDC) based on APRIL, a TNF superfamily ligand with intrinsic dual specificity for BCMA and TACI. Our design strategy was guided by structural analysis of the APRIL crystal structure (Fig. 1A). As native APRIL forms a homotrimer that enhances receptor-binding avidity and promotes efficient cellular internalization, maintaining this trimeric architecture was a key priority. During initial expression screening, we observed low yields of soluble human APRIL in mammalian cell systems. Notably, murine APRIL shares ∼85% sequence identity with its human counterpart within the TNF homology domain, with divergence localized outside the BCMA/TACI binding site. We therefore utilized murine APRIL for proof-of-concept LDC studies. In this study, we engineered a hexavalent fusion protein by linking three murine APRIL monomers in tandem *via* GGGGS flexible linkers, followed by fusion to the Fc domain of human IgG1 (Fig. 1B). For generating APRIL-LDCs by cysteine‒maleimide conjugation, we introduced a single-point mutation at Ser107—a distal site relative to the BCMA/TACI binding interface—substituting serine with cysteine (S107C) in the APRIL structure (Fig. 1B). An anti-BCMA monoclonal antibody (J6M0) was used as a control.

The structural integrity of the fusion protein was validated by reducing SDS-PAGE under denaturing conditions (MW = 75 kDa, Supporting Information Fig. S1A). ELISA confirmed that murine APRILFc binds to both human BCMA and human TACI, with a stronger affinity observed for BCMA (Fig. S1B). To validate binding specificity, flow cytometry analysis demonstrated that both APRILFc and anti-BCMA antibody (BCMAab) exhibited binding capability to cell lines with differential antigen expression profiles (Fig. 1C and D). Notably, APRILFc displayed superior binding affinity compared to the anti-BCMA antibody in both RPMI8226 cells (BCMA^+^/TACI^+^) and Daudi cells (BCMA^‒^/TACI^+^) (Fig. S1C), suggesting enhanced dual-target engagement through its APRIL-mediated recognition mechanism.

### 3.2 Endocytosis capability of APRILFc

**Figure 2:**
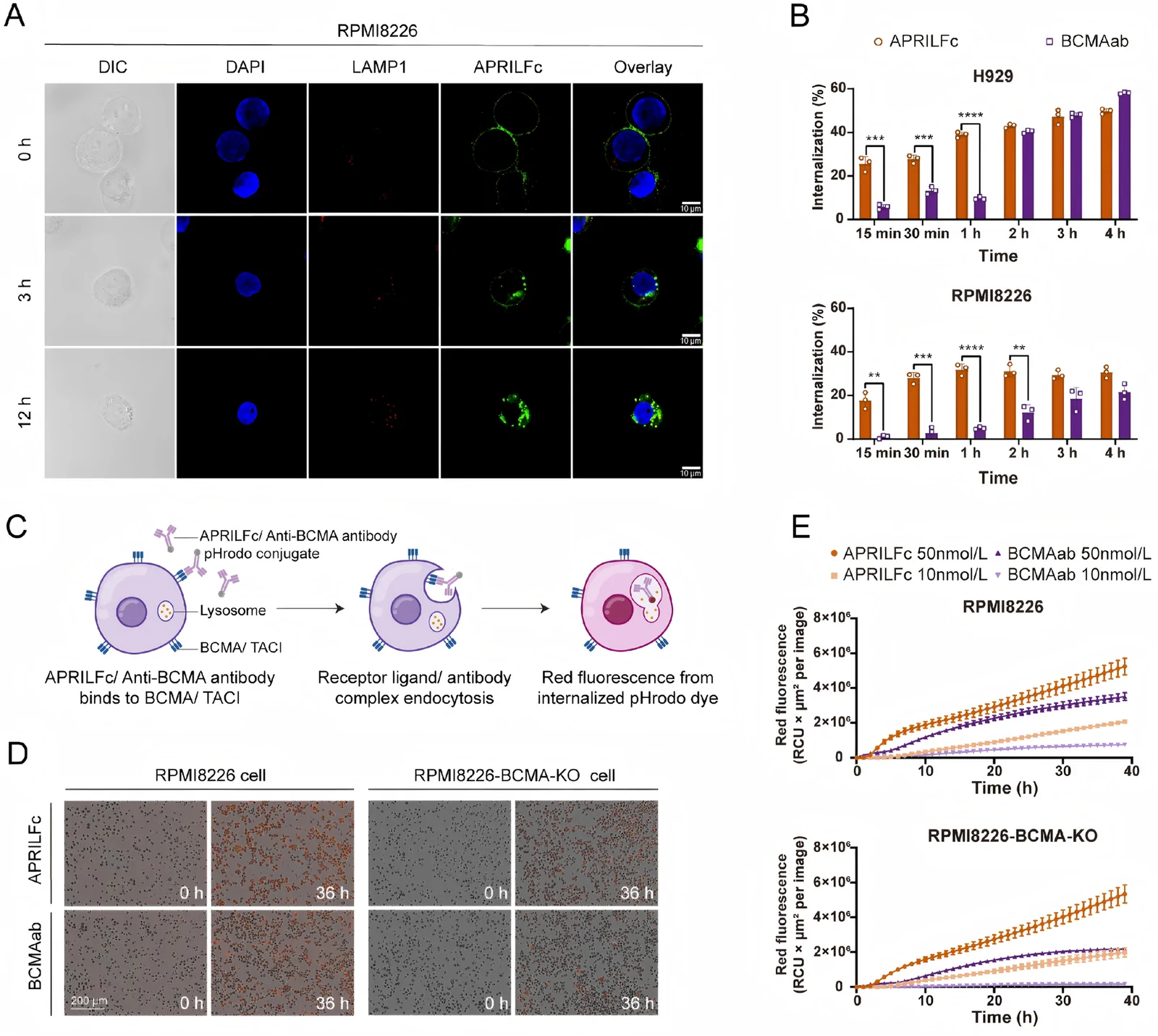
The rapid internalization and lysosome transport ability of APRILFc. (A) The internalization and lysosomal localization of APRILFc in RPMI8226 cells by laser-scanning confocal microscopy. Blue fluorescence: DAPI, red fluorescence: Cy3, green fluorescence: Cy5. Scale bars represent 10 μm. (B) The internalization percentages of APRILFc and BCMAab were detected by flow cytometry. RPMI8226 and H929 cells were incubated with APRILFc or BCMAab at 37 °C for different durations. (C) Illustration of the internalization of APRILFc-pHrodo and BCMAab-pHrodo into MM cells. After binding to cell surface antigens with APRILFc-pHrodo or BCMAab-pHrodo, the complexes undergo endocytosis and fuse in lysosomes, emitting red fluorescence. (D) 50 nmol/L APRILFc-pHrodo or BCMAab-pHrodo was added to RPMI8226 or RPMI8226-BCMA-KO cells and analyzed using live-cell imaging. Scale bars represent 200 μm. (E) Quantification of fluorescence as red calibrated unit (RCU) over time after the APRILFc-pHrodo or BCMAab-pHrodo co-cultured with tumor cells. For (B, E), data are shown as mean ± SD (*n* = 3). Data were analyzed by unpaired two-tailed Student's *t*-test. ∗*P* < 0.05; ∗∗*P* < 0.01; ∗∗∗*P* < 0.001; ∗∗∗∗*P* < 0.0001.

Rapid internalization is a key prerequisite for both antibody‒drug conjugates (ADCs) and LDCs to efficiently deliver cytotoxic payloads into tumor cells. To evaluate the internalization and lysosomal trafficking of the APRILFc fusion protein, we conducted laser scanning confocal microscopy in RPMI8226 cells. Upon confirmation of APRILFc surface binding at 4 °C, cells were incubated at 37 °C to initiate internalization. APRILFc exhibited rapid uptake, with intracellular signals progressively increasing over time and co-localizing with lysosomal markers, confirming its efficient endocytosis and subsequent trafficking to lysosomes (Fig. 2A). Subsequently, we compared the internalization efficiency between BCMAab and APRILFc. Flow cytometric analysis demonstrated significantly faster internalization rates of ligand fusion proteins compared to antibodies in both high-BCMA-expressing H929 cells and moderate-BCMA-expressing RPMI8226 cells. In H929 cells, APRILFc reached ∼25% internalization within 15 min, while the antibody achieved only ∼5%. After 3 h, both reached ∼48%, but APRILFc exhibited markedly superior kinetics (Fig. 2B). In RPMI8226 cells, the difference was even more pronounced: APRILFc internalized ∼17% within 15 min, compared to only ∼1% for the BCMAab. To investigate the impact of BCMA and TACI receptors on the endocytosis of APRILFc, we compared the internalization efficiency in RPMI8226 cells versus RPMI8226-BCMA-KO cells at identical time points. Flow cytometric analysis at the 2-h time point revealed an internalization efficiency of approximately 17.36% in wild-type RPMI8226 cells, compared to only 5.01% in BCMA-knockout cells (Supporting Information Fig. S2A). These results indicate that concurrent engagement of both receptors enhances the endocytic efficiency of APRILFc. Quantitative analysis suggested the relative contributions of BCMA and TACI to this process are approximately 71% and 29%, respectively, highlighting the dominant role of the BCMA receptor. To further elucidate the endocytic mechanism, cells were pre-treated with various trafficking inhibitors prior to the addition of APRILFc and subsequently assessed by flow cytometry (Fig. S2B). The endocytic activity in RPMI8226 cells was significantly attenuated in the presence of clathrin inhibitors (chloroquine, chlorpromazine). Similarly, endocytosis in RPMI8226-BCMA-KO cells was impaired by a macropinocytosis inhibitor (amiloride hydrochloride) and the clathrin inhibitor Chlorpromazine. In contrast, inhibitors targeting lipid raft (methyl-*β*-cyclodextrin) and caveolae (nystatin) had minimal effects on both cell types. Collectively, these findings demonstrate that clathrin-mediated endocytosis and macropinocytosis are primarily involved in the receptor-mediated internalization of APRILFc. To dynamically track lysosomal trafficking, we conjugated APRILFc and BCMAab to a pH-sensitive fluorophore (pHrodo Red) that activates in acidic compartments (Fig. 2C). Real-time live-cell imaging revealed that the APRILFc-pHrodo exhibited superior internalization kinetics compared to the antibody-pHrodo (Fig. 2D). Quantitative analysis further demonstrated significantly higher internalization rates for the APRILFc at matched time points in both RPMI8226 wild-type and BCMA-knockout (RPMI8226-BCMA-KO) cell lines (Fig. 2E). These findings collectively demonstrate that APRILFc undergoes rapid receptor-mediated internalization and directed lysosomal trafficking, highlighting its suitability as a delivery vehicle for LDC-based cancer therapeutics.

### 3.3 *In vitro* antitumor activity of APRILFc-MMAE

**Figure 3:**
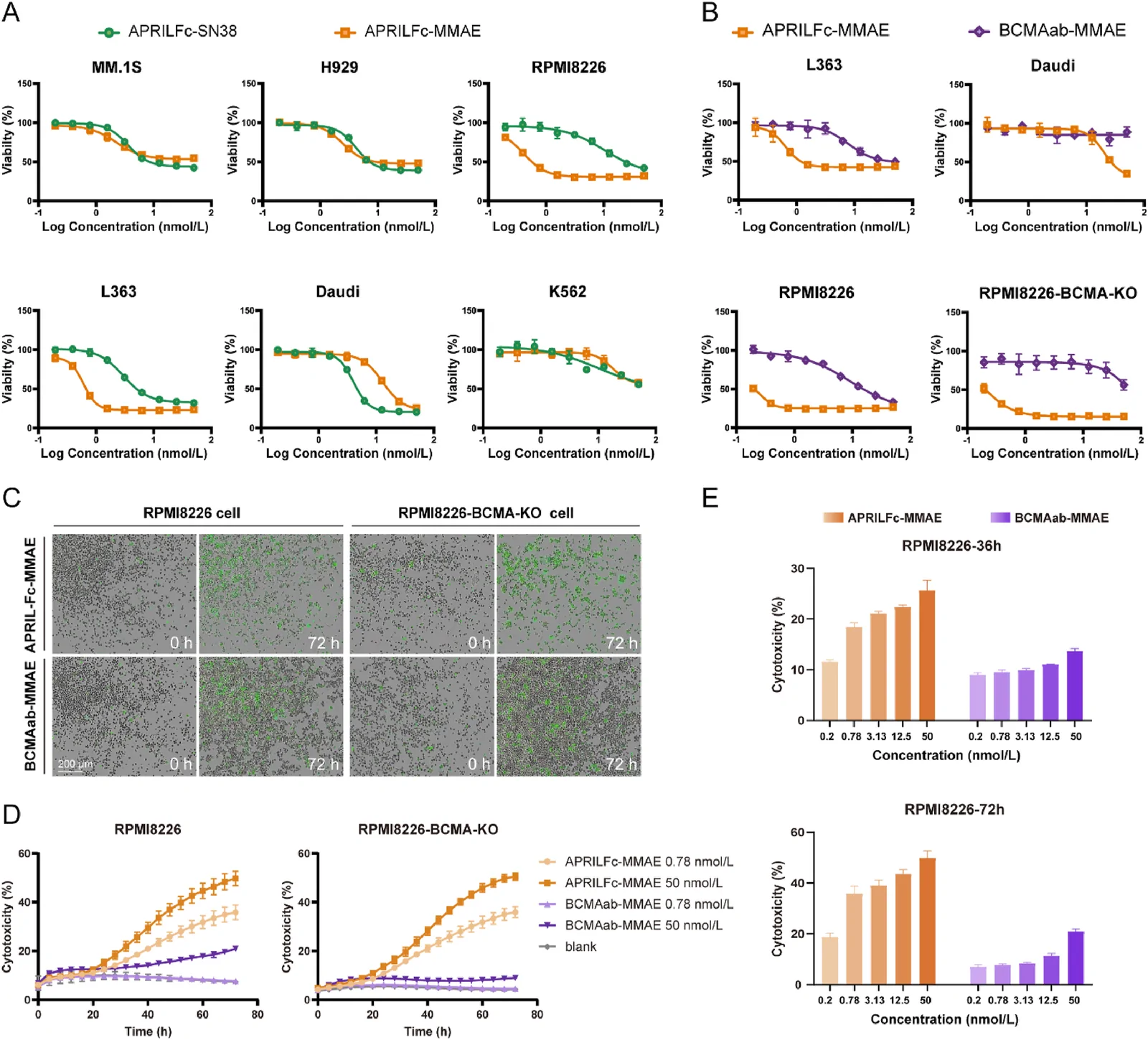
*In vitro* cytotoxicity of APRIL-LDC. (A, B) Cytotoxicity of APRILFc-SN38, APRILFc-MMAE and BCMAab-MMAE to different tumor cell lines was assessed using a CCK-8 assay. (C) RPMI8226 cells and RPMI8226-BCMA-KO cells were treated with APRILFc-MMAE or BCMAab-MMAE at the indicated concentration. Cell death was indicated by green fluorescence areas using live-cell imaging. Scale bars represent 200 μm. (D, E) Quantification of cytotoxicity expressed as the ratio of green fluorescence area to phase area over time after co-incubation with tumor cells. Data are shown as mean ± SD (*n* = 3).

To evaluate the antitumor potential of LDCs derived from APRILFc, we assessed the cytotoxicity of APRIL-LDCs using SN38 or monomethyl auristatin E (MMAE) as payload across a panel of cell lines with differential expression levels of BCMA and TACI. LDCs were synthesized *via* maleimide-thiol conjugation between cysteine residues and toxin-bearing linkers. The integrity and purity of the prepared LDCs were confirmed by SDS-PAGE (Supporting Information Fig. S3A). Both APRILFc-SN38 and APRILFc-MMAE exhibited potent, dose-dependent cytotoxicity in BCMA^+^ and/or TACI^+^ cell lines, while showing no effect in BCMA^−^/TACI^−^ K562 cells, demonstrating target specificity (Fig. 3A). The half-maximal inhibitory concentration (IC_50_) values of both LDCs ranged from low to sub-nanomolar levels in sensitive cell lines (Fig. 3A). Notably, the MMAE conjugate demonstrated superior potency in MM.1S, RPMI8226 and L363 cell lines compared to SN38, prompting its selection for subsequent studies as a proof-of-concept for LDC.

To directly compare the *in vitro* efficacy of ADCs and LDCs, we assessed cytotoxicity of APRILFc–MMAE and anti-BCMA antibody–MMAE (BCMAab–MMAE) in multiple myeloma models (Fig. 3B). APRILFc-MMAE exhibited markedly enhanced cytotoxicity relative to the ADC counterpart in both RPMI8226 and L363 cell lines (BCMA^+^/TACI^+^) with IC_50_ values of 0.26 *vs.* 8.98 nmol/L and 0.64 *vs.* 7.85 nmol/L. In BCMA-low-expressing or BCMA-knockout models (Fig. S3B and S3C) (Daudi and RPMI8226-BCMA-KO cells), BCMAab-MMAE showed minimal efficacy, whereas APRILFc-MMAE retained robust *in vitro* cytotoxicity with IC_50_ values of 20.33 and 0.21 nmol/L, highlighting its dual-targeting advantage through TACI engagement. Elevated levels of soluble BCMA (sBCMA) are present in the serum of patients with multiple myeloma. To model the impact of sBCMA on the antitumor potency of LDCs and ADCs, cells were treated with varying concentrations of sBCMA (0, 10, 100, and 1000 μg/mL) together with different concentrations (5 and 20 nmol/L) of either APRILFc-MMAE or BCMAab-MMAE. The cytotoxic activity of APRILFc-MMAE was only marginally attenuated by the presence of sBCMA across different cell lines, remaining largely comparable to its baseline potency and demonstrating less susceptibility to sBCMA interference than BCMAab-MMAE (Supporting Information Fig. S4A). Given that both BCMA and TACI are expressed on normal immune cells, particularly on terminally differentiated B cells, we assessed the potential on-target off-tumor toxicity. Binding assays confirmed that APRILFc-MMAE could bind to both peripheral blood mononuclear cells (PBMCs) and isolated B cells. However, it did not exhibit significant cytotoxicity against these normal cells, indicating a favorable safety profile for APRILFc-MMAE towards normal immune cells (Fig. S4B and S4C). To further compare the cytotoxic kinetics and antitumor efficacy of ADCs and LDCs, we performed real-time live-cell imaging using Incucyte on RPMI8226 and RPMI8226-BCMA-KO cells. Quantification of fluorescence signals from dead cell staining demonstrated that APRILFc-MMAE induced significantly faster and more pronounced tumor cell killing compared to BCMAab-MMAE (Fig. 3C‒E). Importantly, in the BCMA-escape model (RPMI8226-BCMA-KO cells), APRILFc-MMAE maintained full cytotoxic efficacy, whereas BCMAab-MMAE was largely ineffective, underscoring the therapeutic advantage of dual-targeting LDCs in circumventing antigen escape and improving therapeutic resilience in MM. Given that APRIL is known to promote myeloma cell survival, we further evaluated the safety profile of APRILFc by co-cultivating various multiple myeloma cell lines with the molecule and assessing cell proliferation *via* CCK-8 assay (Supporting Information Fig. S5A). The results demonstrated that even at elevated concentrations, a 72-h incubation with APRILFc did not elicit a significant proliferative response. Subsequently, we analyzed the activation status of key signaling pathways following short-term (2-h) exposure. Western blot analysis revealed that both APRILFc and human APRIL treatment induced rapid phosphorylation of ERK1/2. In contrast, under the same conditions, no significant upregulation in the phosphorylation of p65, a key effector of the NF-*κ*B pathway, was observed (Fig. S5B).

### 3.4 APRILFc-MMAE induces cell apoptosis and cycle arrest

**Figure 4:**
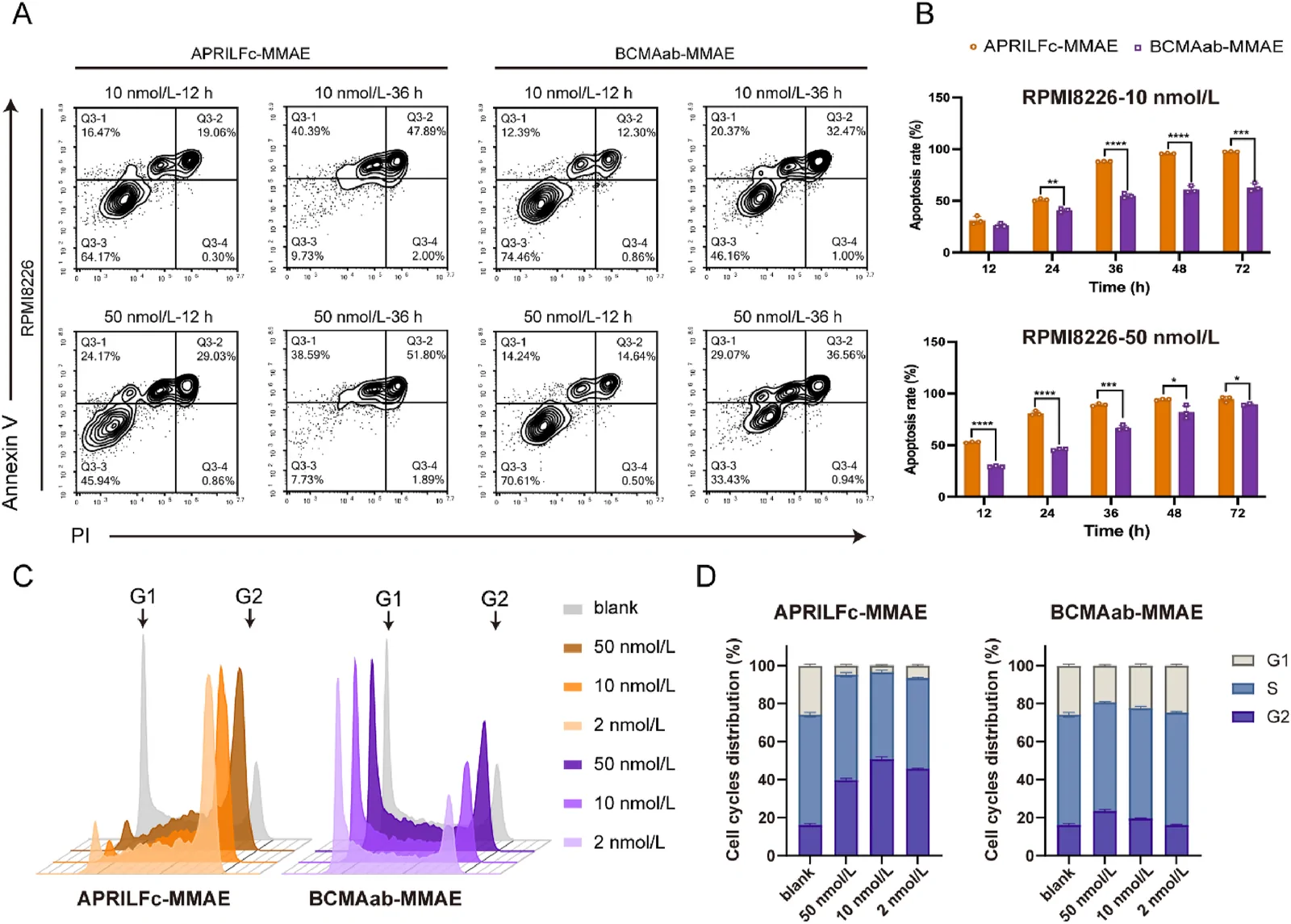
APRIL-LDC induces apoptosis and cell-cycle arrest. (A, B) Apoptosis analysis of RPMI8226 cells after treatment with APRILFc-MMAE or BCMAab-MMAE at the indicated concentration for 12 and 36 h (A), and aggregate data for 12, 24, 36, 48 and 72 h (B). (C, D) Cell-cycle phase distribution of RPMI8226 cells after treatment with APRILFc-MMAE or BCMAab-MMAE at the indicated concentration for 24 h (C), and quantitative analysis (D). For (B, D), data are shown as mean ± SD (*n* = 3). Data were analyzed by unpaired two-tailed Student's *t*-test. ∗*P* < 0.05; ∗∗*P* < 0.01; ∗∗∗*P* < 0.001; ∗∗∗∗*P* < 0.0001.

To elucidate the cytotoxic mechanism of APRILFc-MMAE, we conducted apoptosis and cell cycle analysis in RPMI8226 cells. Cells were treated with APRILFc-MMAE or BCMAab-MMAE at concentrations of 10 and 50 nmol/L for various time points. Apoptosis rates were assessed by flow cytometry and Incucyte live-cell imaging. Both conjugates exhibited concentration- and time-dependent increases in apoptotic cell populations. Notably, APRILFc-MMAE demonstrated superior apoptotic kinetics, inducing significantly higher percentages of apoptotic cells at earlier time points compared to BCMAab-MMAE at equivalent concentrations (Fig. 4A and B, Supporting Information Fig. S6A and S6B). At 36 h, 10 nmol/L APRILFc-MMAE led to 87% apoptosis, compared to only 53% for BCMAab-MMAE at the same concentration. To further investigate effects on cell proliferation, we also examined cell cycle progression by DNA content analysis in RPMI8226 cells treated with varying concentrations of APRILFc-MMAE or BCMAab-MMAE. In BCMAab-MMAE-treated cells, flow cytometry revealed a concentration-dependent reduction in G1 DNA content and a concomitant increase in G2 DNA content (Fig. 4C and D). In contrast, APRILFc-MMAE induced sustained G2/M phase accumulation across all tested doses, with no significant concentration-dependent shifts in G1-phase populations. These findings collectively suggest that APRILFc-MMAE exerts potent antitumor effects through dual mechanisms of apoptosis induction and G2/M cell cycle arrest, achieving superior efficacy at lower doses compared to its monoclonal antibody-based counterpart.

### 3.5 APRILFc-MMAE inhibits tumor growth *in vivo*

**Figure 5:**
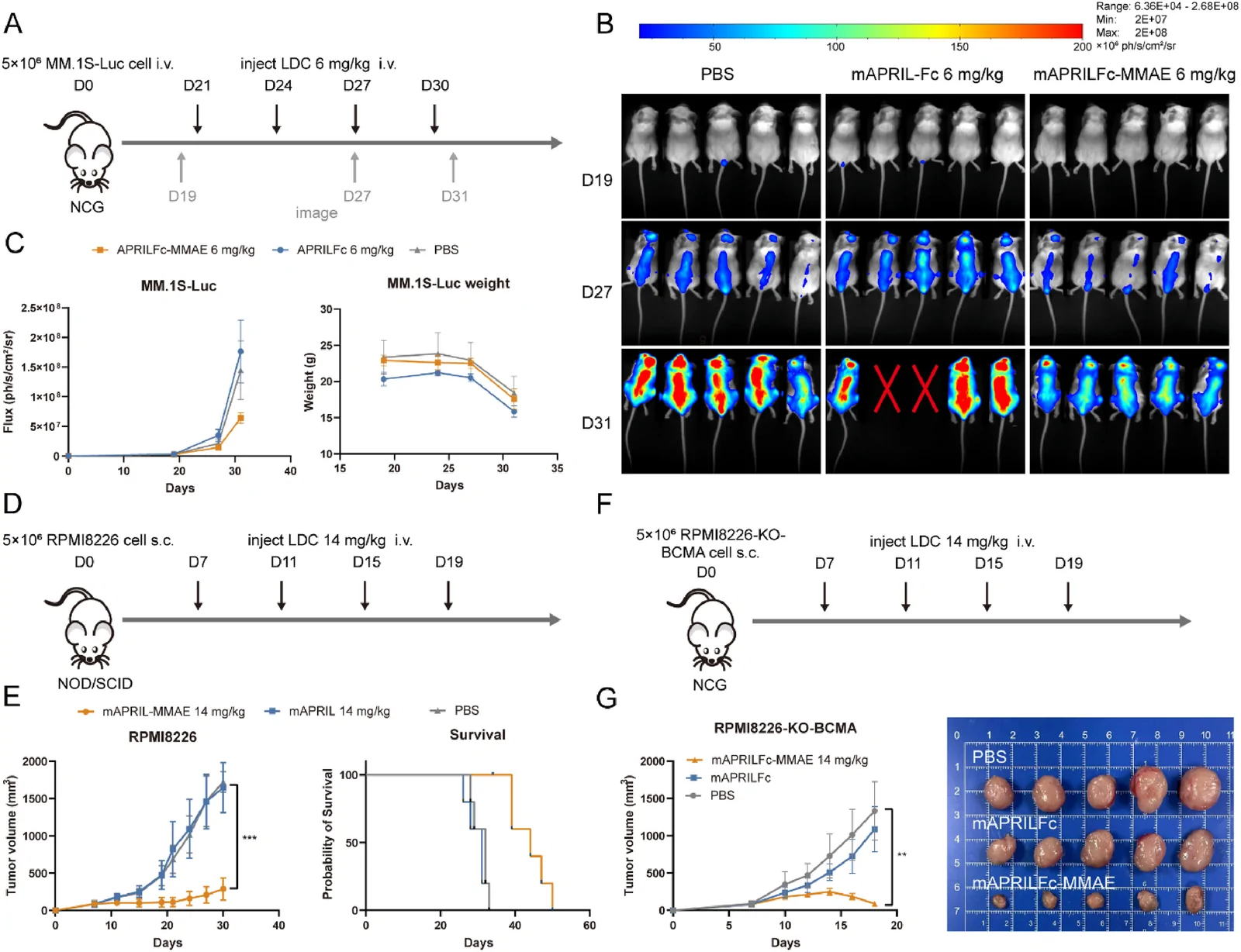
APRIL-LDC activity *in vivo*. (A) Study design of the *in vivo* experiment in NCG mice injected with MM.1S-Luc cells. (B, C) NCG mice were intravenously injected with luciferase-expressing MM.1S cells. On Day 21, mice were treated with PBS, APRILFc or APRILFc-MMAE through tail vein injection. Tumor progression was monitored *via* bioluminescence imaging on the indicated days, Luminescence intensity was measured as radiance (photons/s/cm^2^/sr) (B), with aggregate data (C). (D) Study design of the *in vivo* experiment in NOD/SCID mice injected with RPMI8226 cells. (E) Growth curves and survival curves of RPMI8226 cell-derived xenograft tumors under different treatments. (F) Study design of the *in vivo* experiment in NCG mice injected with RPMI8226-BCMA-KO cells. (G) Growth curves and tumor images of RPMI8226-BCMA-KO cell-derived xenograft tumors under different treatments. For (C, E, G), data are shown as mean ± SD (*n* = 5). To compare the difference in activity between control and the treatment group, data were analyzed by two-way ANOVA with Dunnett's post-test analysis. For (C), differences between survival curves were analyzed by log-rank test. ∗*P* < 0.05; ∗∗*P* < 0.01; ∗∗∗*P* < 0.001; ns, no significance compared with the PBS control.

To assess *in vivo* antitumor efficacy of APRILFc-MMAE, NCG mice were intravenously injected with 5 × 10^6^ MM.1S-Luc cells that stably expressing luciferase. Tumor engraftment was confirmed by bioluminescence imaging (BLI), after which mice received four doses of APRILFc–MMAE (Fig. 5A). Compared to PBS and APRILFc control groups, APRILFc-MMAE demonstrated a significant reduction in tumor burden (Fig. 5B and C), as quantified by decreased total flux (photons/s/cm^2^/sr) in longitudinal BLI measurements.

To further validate the antitumor efficacy of APRILFc-MMAE, we established a subcutaneous xenograft model by inoculating 5 × 10^6^ RPMI8226 cells into the flanks of NOD/SCID mice. Seven days post-inoculation, we initiated treatment when tumors reached an average volume of 80 mm^3^ (calculated as 0.5 × length × width^2^) (Fig. 5D). Compared with the PBS and APRILFc group, APRILFc-MMAE significantly inhibited the growth of RPMI8226 xenografts (*P* < 0.0001) and prolonged overall survival in treated mice (Fig. 5E and F). Histopathological (HE) staining of major organs, including the heart, liver, spleen, lungs, and kidneys, was performed on mice from the PBS, APRILFc, and APRILFc-MMAE treatment groups. Comparative analysis revealed no apparent drug-related pathological lesions in the APRIL-LDC treatment group compared with the control groups (Supporting Informatin Fig. S7A). Furthermore, key biochemical indices were assessed. In mice treated with APRIL-LDC, markers of liver function (ALT, AST) and renal function (BUN, CRE) showed no biologically significant alterations compared to the PBS control group (Fig. S7B). Collectively, these data indicate that administration of APRIL-LDC at its effective therapeutic dose did not induce observable hepatorenal toxicity.To further investigate the antitumor activity of LDCs in the context of antigen escape, we evaluated APRILFc-MMAE efficacy using RPMI8226-BCMA-KO cells in a subcutaneous xenograft model. NCG mice were inoculated with RPMI8226-BCMA-KO cells, and experimental procedures were identical to the previous cohort (Fig. 5G). Notably, APRILFc-MMAE effectively suppressed tumor growth in this BCMA-escape model (*P* < 0.0001) and prolonged overall survival in treated mice, demonstrating its ability to overcome antigen loss through TACI-mediated targeting (Fig. 5F and G).

To evaluate the *in vivo* safety profile of the drug, particularly its toxicity toward B cells, immunocompetent Balb/c mice were employed. Serum levels of IgA and IgM were measured 24 h post-administration to serve as pharmacodynamic markers for B-cell function and viability (Fig. S7C). The results demonstrated no observable depletion of normal plasma cells. Specifically, mice in the APRILFc-MMAE treatment group exhibited no statistically significant decrease in serum levels of either IgA or IgM. A modest, non-significant increase in serum IgA was noted in the APRILFc-MMAE group compared to the PBS control group (*P* = 0.2261).To evaluate the pharmacokinetic profile, BALB/c mice received a single intravenous dose (10 mg/kg) of the test articles *via* tail vein injection. Blood samples were collected at specified time points (30 min, 1, 2, 4, 8, 24, and 48 h) post-administration for serum isolation. The serum concentrations of both APRILFc and APRILFc-MMAE were determined to establish concentration–time curves (Fig. S7D), and key pharmacokinetic parameters were simulated using DAS 2.0 software (Supporting Information Table S1). The results revealed a rapid initial distribution phase within the first 2 h, followed by a stable maintenance phase at lower concentrations that persisted for an extended duration, with detectable drug levels remaining at 48 h. The half-lives of APRILFc and APRILFc-MMAE, as calculated by DAS 2.0, were 157.790 ± 6.441 and 147.585 ± 14.687 h, respectively. These favorable pharmacokinetic characteristics are comparable to many therapeutic monoclonal antibodies and support sustained antitumor efficacy.

## 4 Discussion and conclusions

BCMA-targeted therapies have become a cornerstone in the treatment of multiple myeloma, with several BCMA-directed immunotherapies receiving regulatory approval^30^, ^31^, ^32^. However, clinical observations reveal that CAR-T cell therapies targeting BCMA are frequently associated with antigen escape mechanisms, particularly through BCMA receptor loss, which compromises therapeutic efficacy and correlates with disease relapse^24^^,^^33^. To address this “antigen drift” phenomenon in MM target therapy and enhance tumor antigen density on malignant plasma cells, APRIL was utilized as part of CAR to simultaneously engage BCMA and TACI receptors^28^. This approach aimed to overcome the limitations of single-target BCMA CAR-T therapies that are prone to relapse due to antigen escape mechanisms. Notably, in the Phase I clinical trial (NCT03237804, AUTO2), this dual-targeting strategy demonstrated suboptimal clinical outcomes in MM patients, with only 45.5% of treated patients achieving objective responses^34^^,^^35^. Subsequent mechanistic investigations revealed structural instability and folding abnormalities in the surface-expressed APRIL components, which substantially reduced binding avidity to target antigens and consequently diminished therapeutic efficacy^35^.

To address the structural complexity and instability of APRIL surface presentation while leveraging its inherent advantages of natural bispecificity and high-affinity antigen binding, we developed a novel ligand‒drug conjugate platform based on APRIL trimerization. By integrating APRIL's trimeric architecture with IgG Fc domains, we engineered a stable fusion protein (designated APRILFc) capable of conjugating cytotoxic agents such as monomethyl auristatin E (MMAE) and topoisomerase I inhibitor SN-38. This design enables targeted toxin delivery to MM tumor cells through dual BCMA/TACI receptor engagement. Compared to conventional BCMA-targeting ADCs (*e.g.*, belantamab mafodotin), our APRILFc-MMAE not only exhibits superior or comparable potency in BCMA-positive models, but its fundamental advantage lies in the ability to co-engage TACI. This mechanism retains robust cytotoxicity even in models with low or absent BCMA expression, thereby mitigating a fundamental mechanism of primary and secondary resistance driven by BCMA antigen loss.

To address tumor heterogeneity and drug resistance, bispecific antibodies (BsAbs) have emerged as a class of therapeutics capable of simultaneously binding two distinct target molecules or cells. Building upon this technology, bispecific antibody‒drug conjugates (BsADCs) have become an emerging research focus and are considered a promising strategy for enhancing antitumor efficacy. A representative example is the BsADC MEDI4276, which targets different epitopes on HER2 and induces the formation of large receptor-antibody clusters on the cell surface. This mechanism facilitates accelerated internalization kinetics and enhanced lysosomal trafficking compared to trastuzumab or the parental 39S antibody. Furthermore, MEDI4276 demonstrated significant antitumor activity in mouse xenograft models of refractory HER2^+^ breast cancer (*e.g.*, JIMT-1 and NCI-N87) as well as in patient-derived xenograft (PDX) models with HER2-low heterogeneity^36^. Currently, most dual-targeting antibodies are constructed through the fusion expression or combination of two distinct antibodies^37^, a process that increases production complexity and often results in heterogeneous mixtures. “Two-in-one” single-domain bispecific antibodies, though structurally compact, face substantial screening and selection hurdles, prolonging development timelines^38^. In contrast, a native ligand-conjugated drug (LCD) platform offers a streamlined strategy for bispecific targeting by capitalizing on the innate dual-receptor recognition and high binding affinity of endogenous ligands. This approach avoids the complex and costly process of antibody engineering, potentially offering advantages in both production simplicity and enhanced molecular homogeneity. Leveraging the inherent dual-targeting capability and high affinity of native ligands, LDCs exhibit high specificity and potent endocytic activity, enabling rapid tumor cell killing.

Despite these advantages, further optimization is necessary. In our studies, ligand fusion proteins demonstrated lower stability compared to conventional antibodies. This study represents an early-stage discovery effort aimed at providing proof-of-concept for the bispecific LDC platform. Future work will focus on rational protein engineering, including the directed evolution of human APRIL, to enhance ligand stability and improve the pharmacokinetic profile of the LDC, thereby aligning it more closely with the requirements for clinical translation. Future research should focus on enhancing ligand stability through directed evolution and rational protein engineering, with the goal of improving structural robustness and pharmacokinetic profiles. Additionally, as native ligands possess inherent bioactivity, binding to cognate receptors can modulate downstream signaling pathways, leading to either activation or inhibition^39^^,^^40^. Although our current study demonstrated that treatment with APRILFc did not induce significant proliferative activity in multiple myeloma cell lines and failed to activate the full complement of signaling pathways required to drive cell proliferation, the rapid internalization of the LDCs, coupled with prompt payload release, effectively mediated tumor cell apoptosis. However, this study is primarily focused on the short-term antitumor effects of the LDC. We did not investigate potential tumor-promoting effects of APRILFc under prolonged exposure or in more complex microenvironments, such as in the presence of cytokines like BAFF or co-culture with stromal cells. Furthermore, given the heterogeneity in signaling pathways across different multiple myeloma (MM) patient subgroups^41^^,^^42^ and the varying sensitivity of tumor cells to cytotoxic agents, eliminating the signaling capacity of ligands represents a crucial optimization strategy. Engineered ligands should maintain high-affinity dual-receptor binding while abolishing unwanted receptor-mediated signaling, thus enhancing safety and therapeutic precision. The clinical translation of the LDC platform faces several key challenges. These include scaling up the manufacturing process while ensuring the stable production of APRIL-LDC with a homogeneous drug-to-antibody ratio (DAR), and optimizing the long-term stability of the ligand‒linker‒drug complex. From a molecular design perspective, the maleimide-thiol chemistry employed in this proof-of-concept study, while effective, has inherent limitations *in vivo* due to potential retro-Michael reactions^43^. For future clinical development, adopting more stable conjugation strategies is imperative to maximize plasma stability, minimize premature payload release, and ultimately enhance the therapeutic index.

This study elucidates the mechanism underlying the enhanced internalization of APRIL-LDC by integrating genetic knockout models and endocytic blockade. Our data clearly demonstrate that its superior efficiency is not reliant on a single receptor, but stems from its ability to exploit both BCMA and TACI as dual-track internalization pathways. In dual-positive cells, these receptors function synergistically, while TACI alone is sufficient to mediate rapid uptake in TACI-only models. We posit that the innate trimeric structure of APRIL enables efficient cross-linking and clustering of surface BCMA and/or TACI receptors. This multivalent interaction likely facilitates the formation of larger receptor complexes, which more effectively recruit clathrin-coated pits and accelerate vesicular internalization. Although direct visualization of receptor clustering remains a goal for future validation, the current functional data strongly support this model. This ligand-mediated dual-target clustering and cooperative internalization represent a core mechanistic advantage of APRIL-LDC over conventional monospecific ADCs.

It is noteworthy that for any dual-targeting agent, the potential for “on-target, off-tumor” effects requires careful consideration. In our study, APRIL-LDC demonstrated compelling *in vivo* efficacy alongside a favorable safety profile. Specifically, it exhibited no significant cytotoxicity against immune cells in co-culture assays and induced no overt systemic toxicity in mouse models. Although APRIL-LDC targets both BCMA and TACI, the expression of these receptors is predominantly restricted to plasma cells and certain mature B-cell subsets. This restricted expression profile inherently limits the potential scope for APRIL-LDC to elicit on-target, off-tumor toxicity beyond the intended target population. Nevertheless, formal toxicology studies in relevant species with larger cohorts remain essential in future development to fully exclude this possibility.

Furthermore, a strategic approach to enhance the efficacy of BCMA-targeted therapies—including BCMA-ADCs, BCMA-CAR-T cells, and our APRIL-LDC—is combination with a *γ*-secretase inhibitor (GSI). GSIs inhibit the proteolytic shedding of BCMA from the myeloma cell surface, thereby increasing target density and potentially improving drug delivery and cytotoxicity^44^, ^45^, ^46^. Future studies exploring the synergistic potential of APRIL-LDC with GSIs are warranted, as this may provide a validated path to overcome microenvironment-driven resistance. Concurrently, given the potential emergence of BCMA-/TACI- immune escape variants under prolonged dual-targeting pressure, combination strategies with other antigen-targeting agents (*e.g*., against GPRC5D^47^ or FcRH5) or with non-overlapping mechanisms of action (such as immunomodulatory agents or proteasome inhibitors) should be considered. Such multi-pronged therapeutic approaches could be crucial for achieving durable long-term remissions.

In summary, we have developed a ligand‒drug conjugate (LDC) platform leveraging a ligand-centric design to simultaneously target BCMA and TACI. In vitro antitumor activity studies demonstrated that APRILFc-MMAE induces tumor cell apoptosis at an accelerated rate compared to conventional antibody‒drug conjugates (ADCs). Remarkably, cytotoxic activity was retained under conditions of BCMA downregulation, indicating effective TACI-mediated compensation. Furthermore, the rapid internalization kinetics of APRIL minimized membrane retention, reducing nonspecific payload release into the tumor microenvironment (TME). This feature likely contributes to a favorable toxicity profile while preserving therapeutic efficacy. These results establish a proof-of-concept for exploiting native ligands as dual-targeting agents in multitargeted therapeutics, offering enhanced specificity and reduced oncogenic risk in the treatment of multiple myeloma treatment.

## Author contributions

Liqiang Pan: Conceptualization, Supervision and Investigation. Jisheng Wang: Supervision and Resources. Jun He: Supervision and Resources. Linling Zhou: Methodology, Investigation, Writing - Original Draft, Visualizaiton. Yanping Sun: Methodology, Validation, Writing - Review & Editing. Jie Bi: Methodology, Formal analysis, Visualization. Jiaqi Zhao: Formal analysis, Writing - Review & Editing, Visualization. Yinli Xia: Validation, Writing - Original Draft, Validation.

## Conflicts of interest

The authors declare no conflicts of interest.

## Supporting information

The following is the Supporting Information to this article.
