# Supplementary material for: APRIL-driven BCMA/TACI dual-targeted ligand–drug conjugates for selective and potent therapy of multiple myeloma
Source: Acta Pharm Sin B. 2026 Feb 6;16(9):5540–52. doi: 10.1016/j.apsb.2026.02.001 (PMC13590012; doi:10.1016/j.apsb.2026.02.001)
Supplement: Supplementary file 1 [file mmc1.pdf]

Supporting Information for

Original article

APRIL-driven BCMA/TACI dual-targeted ligand–drug conjugates for selective and potent therapy of multiple myeloma

Linling Zhou[a], Yanping Sun[a], Jie Bi[a], Jiaqi Zhao[a], Yinli Xia[b], Jun He[c,*], Jisheng Wang[c,*], Liqiang Pan[a,b,*]

[a]*College of Pharmaceutical Sciences, and Department of Pharmacy, The Second Affiliated Hospital, Zhejiang Univercity, Hangzhou 310058, China*

[b]*School of pharmacy, Hangzhou Medical College, Hangzhou 311399, China*

[c]*The Third Hospital of Mianyang (Sichuan Mental Health center), Mianyang 612000, China*

Received 31 July 2025; received in revised form 28 October 2025; accepted 17 November 2025

*Corresponding authors.

E-mail addresses: panliqiang@zju.edu.cn (Liqiang Pan),
wangjishenglaoshi@163.com (Jisheng Wang), he-001jun@163.com (Jun He).

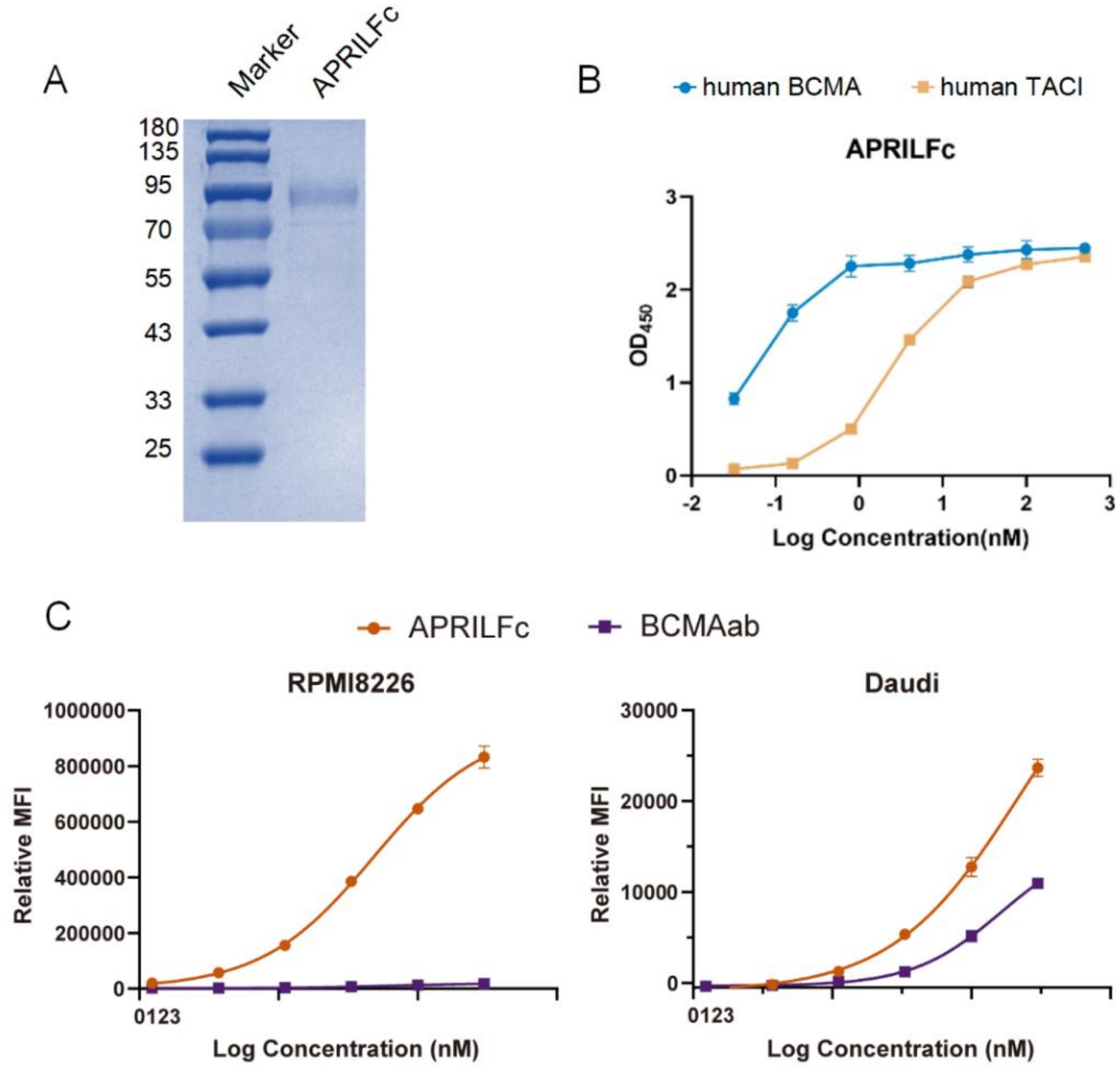

Figure S1 (A) SDS-PAGE gel showing the molecular weights of the reduced APRILFc. (B) Measurement of APRILFc (5 µg/mL) binding affinity to human BCMA or human TACI *via* ELISA. (C) Binding affinity of APRILFc and BCMAab on RPMI8226 and Daudi cells.

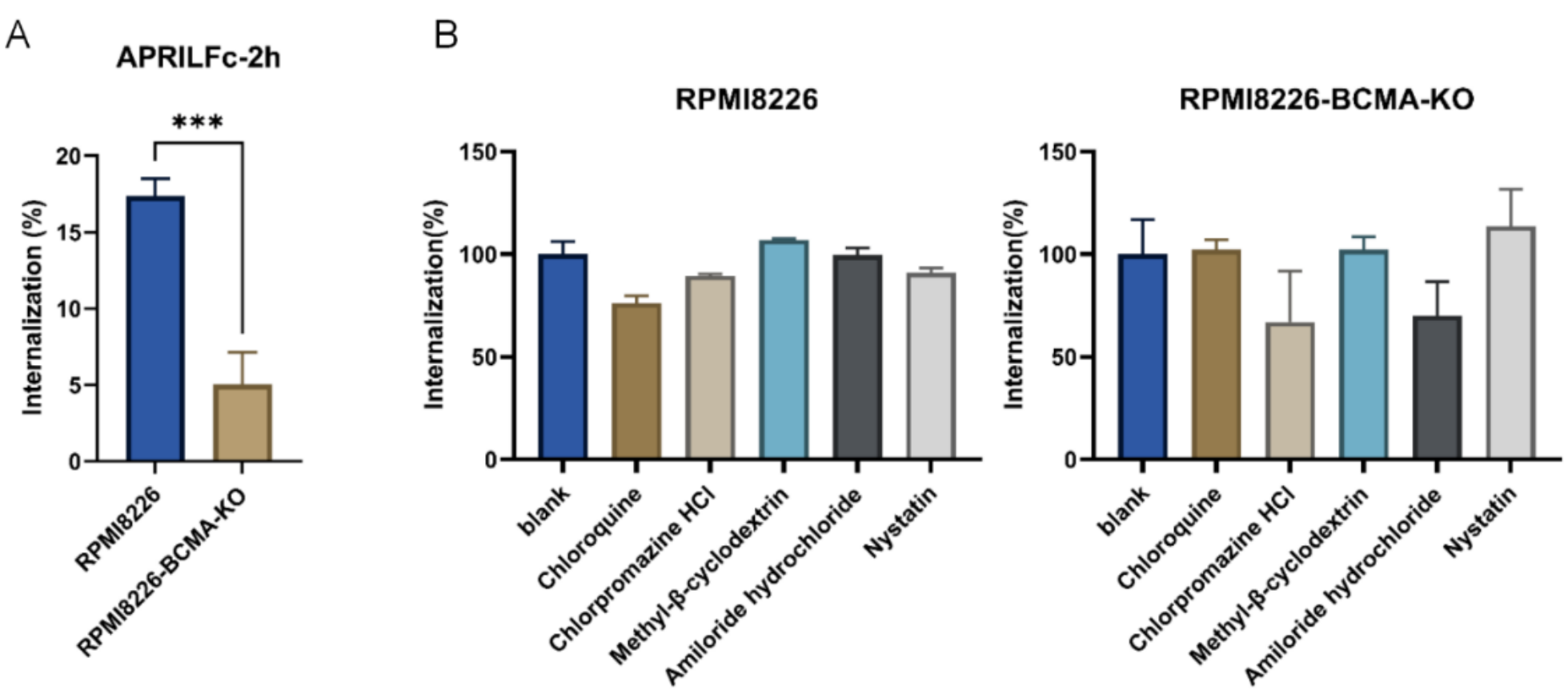

Figure S2 (A) Internalization assay of APRILFc in RPMI8226 and RPMI8226-BCMA-KO cells was performed by flow cytometry following a 2-h incubation at 37 °C. (B) RPMI8226 and RPMI8226-BCMA-KO cells were treated with endocytosis inhibitors

(Chloroquine 500 μmol/L, Chlorpromazine HCl 9 μg/mL, Methyl-β-cyclodextrin 100 μg/mL, Amiloride hydrochloride 100 μg/mL, and Nystatin 10 μg/mL). The percentage of internalization after 2 h was then quantified by flow cytometry, with the blank control group set as 100%.

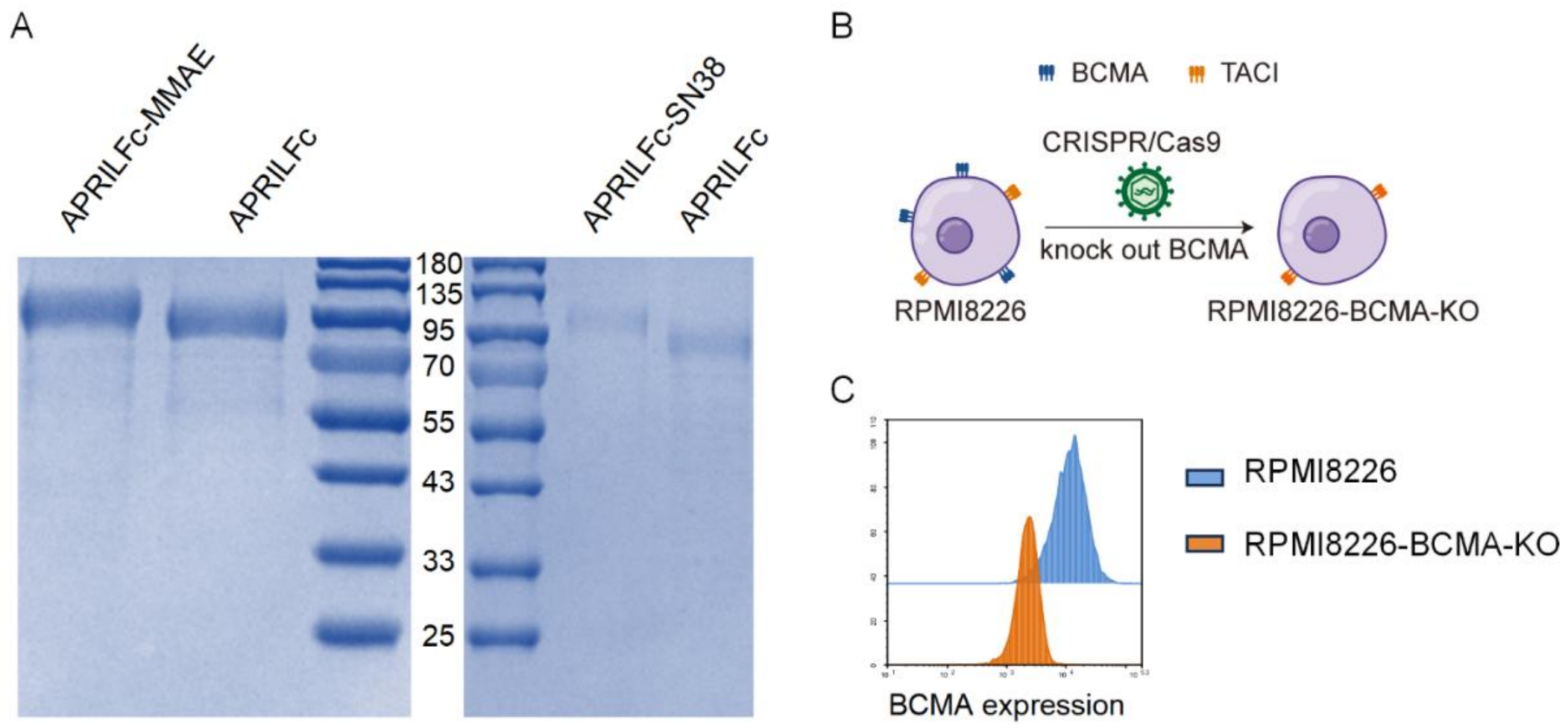

Figure S3 (A) SDS-PAGE gel showing the molecular weights of the reduced APRILFc, LDC-MMAE and LDC-SN38. (B) Schematic diagram of BCMA receptor knockout in RPMI8226 cells using CRISPR/Cas 9 technology. (C) Flow cytometric analysis of BCMA receptor expression levels in the RPMI8226-BCMA-KO cell line.

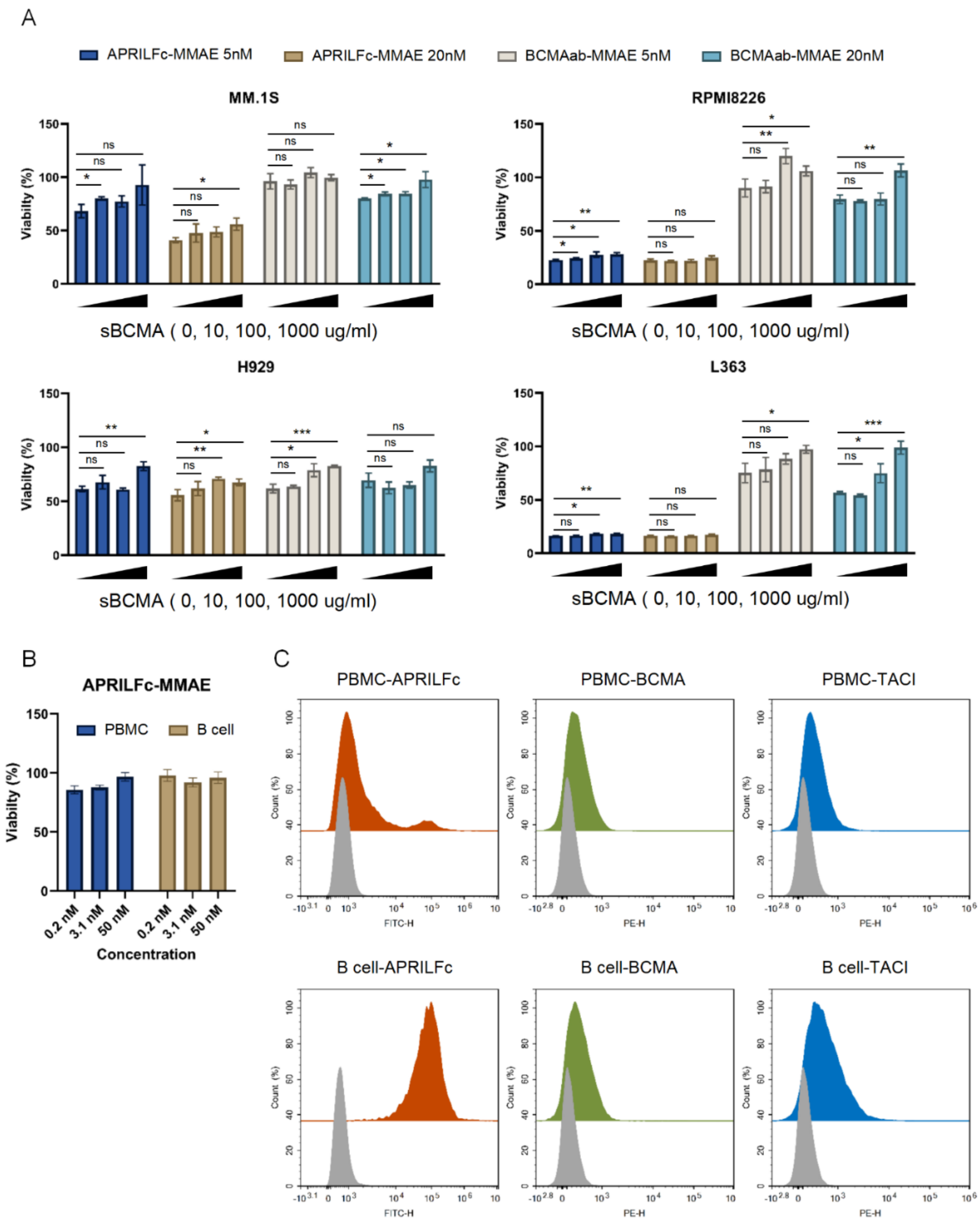

Figure S4 (A) Varying concentrations of sBCMA (0, 10, 100, and 1000 μg/mL) were introduced into cultures of MM.1S, RPMI8226, H929, and L363 cells, along with either 5 or 20 nmol/L of APRILFc-MMAE or BCMAab-MMAE. Cell viability was assessed after 72 h of co-incubation. Data are shown as mean ± SD (n=3). Statistical significance was determined by unpaired two-tailed Student's t-test. *P< 0.05; ** P < 0.01; *** P < 0.001; **** P < 0.0001. (B) Isolated PBMCs and B cells were treated with increasing concentrations of APRILFc-MMAE (0.2, 3.1, and 50 nmol/L) for 72 h, followed by cell viability measurement using the CCK-8 assay. (C) Binding of APRILFc to PBMCs and

B cells, along with cell surface expression of BCMA and TACI, was analyzed by flow cytometry. Histograms are shown as follows: gray line, blank control; red line, APRILFc binding; green line, BCMA expression; blue line, TACI expression.

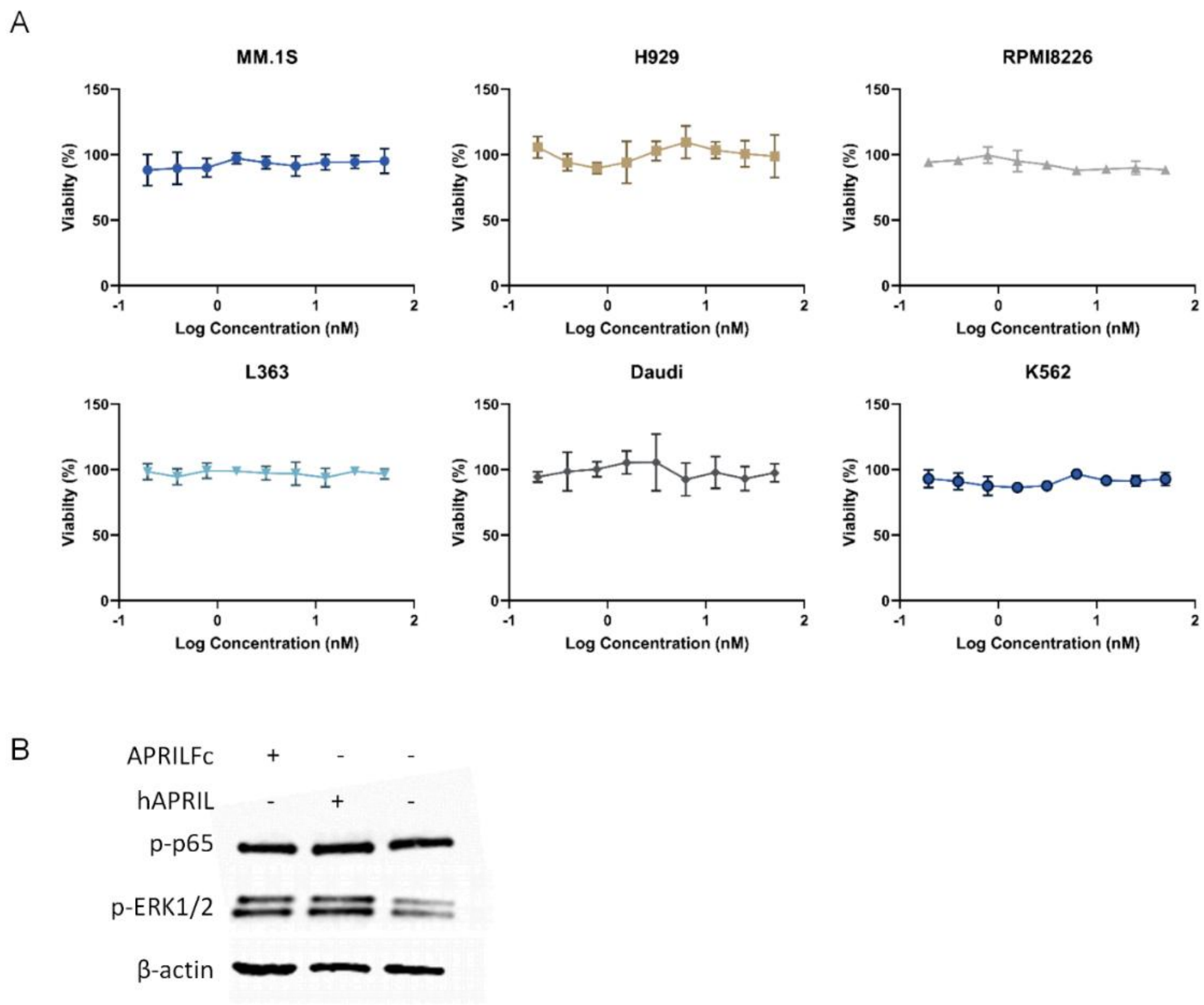

Figure S5 (A) Various multiple myeloma (MM) cell lines were treated with a concentration gradient of APRILFc. Its potential proliferative effect was subsequently assessed *via* CCK-8 assay after 72 h of incubation. (B) MM.1S cells were stimulated with 5 nmol/L APRILFc or human APRIL for 2 h. The phosphorylation levels of key signaling molecules in the ERK1/2 and NF-κB pathways (p-ERK1/2 and p-p65, respectively) were then analyzed by Western blot.

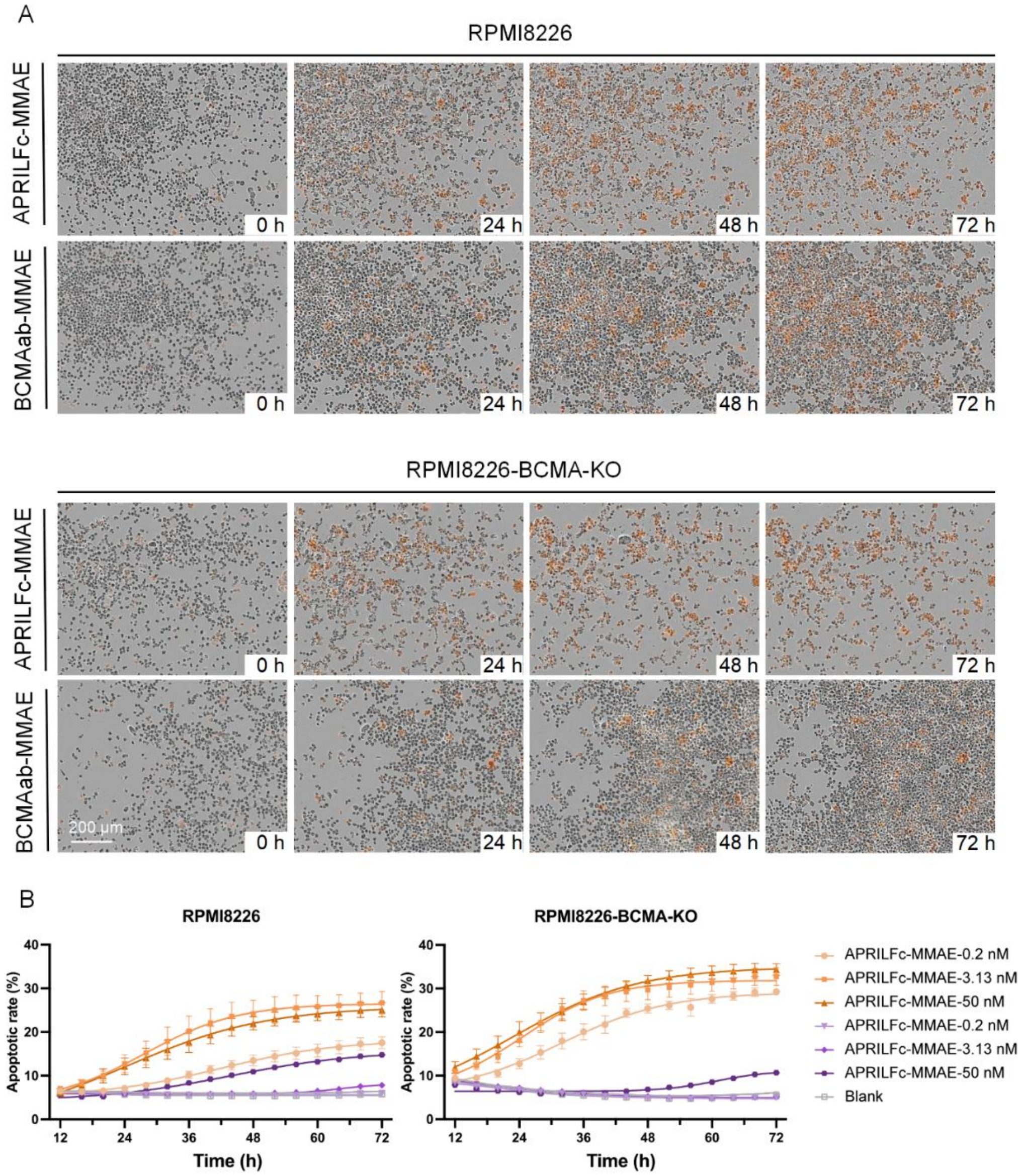

Figure S6 (A) RPMI8226 cells and RPMI8226-BCMA-KO cells were treated with APRILFc-MMAE or BCMAab-MMAE at the indicated concentration. Cell apoptosis was indicated by red fluorescence areas using live-cell imaging. (B) Quantification of apoptosis by red fluorescence areas/ phase areas over time after the APRILFc-MMAE or BCMAab-MMAE co-cultured with tumor cells.

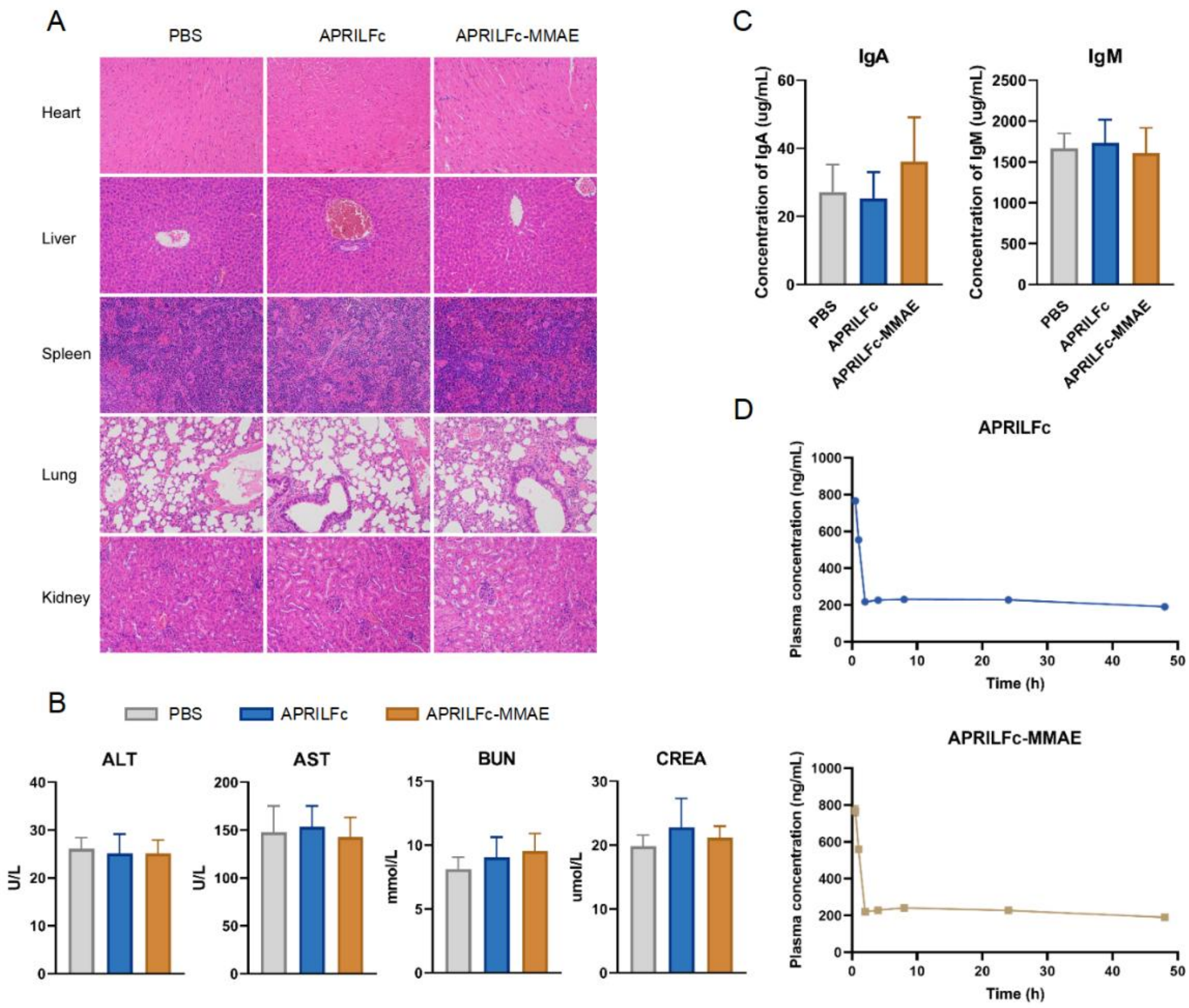

Figure S7 (A) Histopathological assessment of major organs. Heart, liver, spleen, lungs, and kidneys were collected from mice at the experimental endpoint, processed for hematoxylin and eosin (H&E) staining, and examined for potential tissue damage. Representative micrographs are shown at 200× magnification.(B) Analysis of serum biochemical markers for hepatorenal function. Serum was isolated from peripheral blood of mice and concentrations of alanine aminotransferase (ALT), aspartate aminotransferase (AST), blood urea nitrogen (BUN), and creatinine (CREA) were measured to evaluate liver and kidney function. Data are presented as mean ± SD (*n*=5).(C) Evaluation of effects on humoral immunity. Eight-week-old Balb/c mice received a single intravenous dose of the drug (10 mg/kg) *via* the tail vein. Peripheral blood serum was collected 24 h post-administration, and the levels of immunoglobulin A (IgA) and immunoglobulin M (IgM) were quantified using specific ELISA kits. Data are shown as mean ± SD (*n*=5). (D) Pharmacokinetic profiles of APRILFc and APRILFc-MMAE. APRILFc or APRILFc-MMAE (10 mg/kg) was administered to BALB/c mice *via* tail vein injection, and serum concentrations were determined by ELISA. Results are expressed as mean ± SD from 5 mice (*n*=5).

Table S1 Comparison of pharmacokinetic parameters after administering APRILFc or APRILFc-MMAE to mice at 10 mg/kg i.v. (*n*=5).

Pharmacokinetic parameters	APRILFc	APRILFc-MMAE
AUC(0-∞)[a] (μg/L*h)	55235.957±1826.756	53812.303±4180.278
$t_{1/2}$ [b] (h)	157.79±6.441	147.585±14.687
CL[c] (L/h/kg)	0.181±0.006	0.187±0.014

[a] AUC(0-∞): Area under the curve from zero to infinity. [b] $t_{1/2}$: Half-life. [c]CL: Clearance.
